# Fish consumption, cognitive impairment and dementia: an updated dose-response meta-analysis of observational studies

**DOI:** 10.1007/s40520-024-02823-6

**Published:** 2024-08-20

**Authors:** Justyna Godos, Agnieszka Micek, Walter Currenti, Carlotta Franchi, Andrea Poli, Maurizio Battino, Alberto Dolci, Cristian Ricci, Zoltan Ungvari, Giuseppe Grosso

**Affiliations:** 1 Department of Biomedical and Biotechnological Sciences, University of Catania, Catania, Italy; 2 Center for Human Nutrition and Mediterranean Foods (NUTREA), University of Catania, Catania, Italy; 3 Statistical Laboratory, Faculty of Health Sciences, Jagiellonian University Medical College, Kraków, 31-501 Poland; 4 Laboratory of Pharmacoepidemiology and Human Nutrition, Department of Health Policy, Istituto di Ricerche Farmacologiche Mario Negri IRCCS, Milan, 20156 Italy; 5 Italian Institute for Planetary Health (IIPH), Milan, 20124 Italy; 6 Nutrition Foundation of Italy (NFI), Milan, 20124 Italy; 7 Department of Clinical Sciences, Università Politecnica Delle Marche, Ancona, Italy; 8 Research Group on Food, Nutritional Biochemistry and Health, Universidad Europea del Atlántico, Isabel Torres 21, Santander, 39011 Spain; 9 International Joint Research Laboratory of Intelligent Agriculture and Agri-Products Processing, Jiangsu University, Zhenjiang, Jiangsu China; 10 Sustainable Development Department, Bolton Food SpA, Milan, 20124 Italy; 11 Africa Unit for Transdisciplinary Health Research (AUTHeR), North-West University, Potchefstroom, 2531 South Africa; 12 Vascular Cognitive Impairment, Neurodegeneration and Healthy Brain Aging Program, Department of Neurosurgery, University of Oklahoma Health Sciences Center, Oklahoma City, OK USA; 13 Oklahoma Center for Geroscience and Healthy Brain Aging, University of Oklahoma Health Sciences Center, Oklahoma City, OK USA; 14 International Training Program in Geroscience, Department of Public Health, Doctoral College, Semmelweis University, Budapest, Hungary; 15 Stephenson Cancer Center, University of Oklahoma, Oklahoma City, OK USA; 16 Department of Health Promotion Sciences, College of Public Health, University of Oklahoma Health Sciences Center, Oklahoma City, OK USA

**Keywords:** Fish, Dementia, Cognitive status, Alzheimer’s disease, Meta-analysis

## Abstract

**Background:**

Cognitive impairment is projected to affect a preponderant proportion of the aging population. Lifelong dietary habits have been hypothesized to play a role in preventing cognitive decline. Among the most studied dietary components, fish consumptionhas been extensively studied for its potential effects on the human brain.

**Aims:**

To perform a meta-analysis of observational studies exploring the association between fish intake and cognitive impairment/decline and all types of dementia.

**Methods:**

A systematic search of electronic databases was performed to identify observational studies providing quantitative data on fish consumption and outcomes of interest. Random effects models for meta-analyses using only extreme exposure categories, subgroup analyses, and dose-response analyses were performed to estimate cumulative risk ratios (RRs) and 95% confidence intervals (CIs).

**Results:**

The meta-analysis comprised 35 studies. Individuals reporting the highest vs. the lowest fish consumption were associated with a lower likelihood of cognitive impairment/decline (RR = 0.82, 95% CI: 0.75, 0.90, *I*^*2*^ = 61.1%), dementia (RR = 0.82, 95% CI: 0.73, 0.93, *I*^*2*^ = 38.7%), and Alzheimer’s disease (RR = 0.80, 95% CI: 0.67, 0.96, *I*^*2*^ = 20.3%). The dose-response relation revealed a significantly decreased risk of cognitive impairment/decline and all cognitive outcomes across higher levels of fish intake up to 30% for 150 g/d (RR = 0.70, 95% CI: 0.52, 0.95). The results of this relation based on APOE ε4 allele status was mixed based on the outcome investigated.

**Conclusions:**

Current findings suggest fish consumption is associated with a lower risk of cognitive impairment/decline in a dose-response manner, while for dementia and Alzheimer’s disease there is a need for further studies to improve the strength of evidence.

**Supplementary Information:**

The online version contains supplementary material available at 10.1007/s40520-024-02823-6.

## Introduction

Over the last decades, the increase in human lifespan and the growing older population worldwide has changed the epidemiology of diseases leading to a substantial rise in age-related health conditions [1, 2]. Mental and cognitive health have been reported to represent an emerging global issue for elderly individuals worldwide [3]. Specifically concerning cognitive-related conditions, Alzheimer’s disease (AD) and other dementias have been estimated to account for about nearly 60 million cases globally projected to rise 3-fold by 2050 [4]. [5, 6] Diet is nowadays recognized to affect human brain and mental health conditions [7, 8]. Several dietary components, such as healthy fats, certain amino acids and oligopeptides, antioxidant vitamins and phytochemicals [i.e., (poly)phenols] are recognized to play a role in preserving neuron stability and functionality as well as counteracting neuroinflammation [9–11]. Dietary patterns characterized by fish consumption as one of the main sources of protein [i.e., the Mediterranean diet, the Nordic diet, and the Dietary Approach to Stop Hypertension (DASH)] have been consistently associated with lower risk of neurodegenerative conditions [12–14]. Fish has long been studied for its role on human health [15]. Its content in omega-3 polyunsaturated fatty acids (PUFAs) is considered the culprit for the potentially beneficial effects of seafood on mental health [16], while only relatively recently additional attention has been paid to bioactive oligopeptides (bioactive molecules composed of only few amino acids) and their ability to exert direct effects in the brain, demonstrating anti-inflammatory and antioxidant activities [17, 18]. Although the rationale behind the potential beneficial effects of fish intake in neurodegenerative diseases is quite convincing, it is still unclear whether fish consumption per se might play a role in the prevention of cognitive decline and dementia. Two recent meta-analyses explored the role of fish and cognitive outcomes reporting a dose-response association with lower risk of dementia and Alzheimer’s disease [19, 20]: however, the results are outdated, a broader exploration of cognitive outcomes could be further implemented, risk estimates were only provided by weekly intakes, and some missing entries could be integrated. Hence, the aim of the present study was to update current evidence of the association between fish consumption and cognitive decline, impairment, and dementia risk reported in observational studies and provide a summary meta-analysis of the results.

## Methods

The design and reporting of this study followed the Meta-analyses Of Observational Studies in Epidemiology (MOOSE) guidelines (Supplementary Table 1) [21]. The systematic review protocol was registered in the PROSPERO International Prospective Register of Systematic Reviews database (ID: CRD42024501232, at https://www.crd.york.ac.uk/prospero/).

### Search strategy and study selection

To identify potentially eligible studies, a systematic literature search of PubMed and Scopus databases was performed from their inception up to March 2024. The search strategy was based on the combination of the relevant keywords imputed as text words and MeSH terms, related to fish, seafood and shellfish and cognitive outcomes (Supplementary Table 2). Eligibility criteria for the systematic review and meta-analysis were specified using the PICOS approach (Supplementary Table 3). Studies were eligible if they met the following inclusion criteria: (1) conducted on older adults (i.e., mean age > 50 years old) or, more in general, investigating cognitive outcomes occurring at older age; (2) had observational design (cohort studies, cross-sectional studies, case-control studies); (2) reported exposures to habitual fish, seafood, or shellfish consumption assessed through either 24-h recalls, food frequency questionnaires (FFQ), or dietary diaries; (4) investigated cognitive impairment, cognitive decline, and/or any type of dementia (including Alzheimer’s disease) as outcome; and (5) provided probability measures [odds ratios (ORs), relative risks (RRs), or hazard ratios (HRs)] for the cognitive outcomes investigated. Although the systematic search was not language restricted, only English language studies were eligible. Reference lists of all eligible studies were also examined for any additional studies not previously identified. If more than one study reported results on the same cohort, only the study including the larger cohort size, the longest follow-up, or the most comprehensive data was included in the meta-analysis. The systematic literature search and study selection were performed by two independent authors (J.G. and G.G.) and any incongruity was resolved through a discussion and reaching consensus.

### Data extraction and quality assessment

Data from all eligible studies were extracted using a standardized electronic form. The following information was collected: first author name, publication year, study design and location, population age and gender, sample size, details on the assessment method of dietary habits, details on the exposure, details on the assessment method of the outcome of interest, outcome of interest, main findings of the study, measures of association including 95% confidence intervals. The quality of each eligible study was evaluated using the Newcastle-Ottawa Quality Assessment Scale, consisting of 3 domains of quality (selection, comparability, and outcome) and assessing specific study characteristics depending on the type of study design [22]: in general, studies scoring over 5 and 7 points for cross-sectional and prospective studies, respectively, were identified as being of good/high quality. Two investigators extracted the data and assessed the methodological quality independently and any incongruity was resolved through a discussion and reaching consensus.

### Statistical analysis

Various risk measures, such as odds ratios (ORs) and hazard ratios (HRs) under the rare disease assumption were treated approximately equivalent to risk ratios and further all were consistently denoted by RRs. The logarithms of RRs from fully adjusted models were pooled in meta-analysis to compare the risk of cognitive events between extreme categories of fish consumption and to reveal dose-dependent relationships. Cognitive impairment and cognitive decline were deemed as a single outcome because, although not clearly stated in all studies providing such outcomes, they both most likely referred to age-related conditions or early-stage disease. All-type dementias and Alzheimer’s disease were investigated as individual separate endpoints. No further data on other specific types of dementia was available in the included studies. RRs for independent studies reported in the same article (i.e., for NHS and HPFS cohorts), were analyzed as separate estimates. When risk estimates were provided for males, females and both sexes together, the latter were used in the main analyses;. when pooled data by sex was not provided in the original study, risk estimates were first pooled using a fixed effect meta-analysis to obtain the joint RR. Der Simonian and Laird random-effects model was applied in which weights of the studies were calculated as the inverse of the sum of both within- and between-study variance [23]. The differences in the research results included in the meta-analysis reflected by the degree of heterogeneity were assessed by the Cochran’s Q-test and the I² index. For Q-test the level of significance was set at *p* < 0.10 and the value of *I*^*2*^ statistic exceeding 50% was regarded as considerable heterogeneity between studies. A non-linear dose–response meta-analysis was performed only for studies which reported RRs for at least 3 different levels of well-defined fish intake. If the range of fish consumption was not given, the right-unbounded interval was assumed to be the same width as of the adjacent category, while the left-unbounded interval we set to zero. For each category of exposure, the medians, means or midpoints of ranges of daily consumption were extracted directly from the original studies and assigned to the corresponding RRs. When specific quantity of fish intake was not available, daily fish consumption was calculated by multiplying the frequency of consumption (number of serving per day) by the average portion size estimated as 105 g [24, 25]. A dose–response meta-analysis was modeled by restricted cubic splines with the knots at fixed percentiles of the fish intake distribution (10%, 50%, and 90%) [26]. If the distribution of cases and number of participants or person-years was accessible for all categories of fish consumption, we applied the generalized least squares method to estimate trend from summarized dose-response data accounting for the correlation between extracted RRs [27, 28]. Otherwise, a standard technique based on weighted least squares analysis was adapted [27]. The between-study variance-covariance matrices were assessed via multivariate extension of the method of moments to combine all the regression coefficients across studies. A non-linearity was tested by verifying whether the coefficient of the second spline differ from zero. To compare the effects of fish consumption on cognitive function in specific subgroups in which the risk of disease could potentially differ, analyses in strata of APOE genotype (carrying APOE ε4 allele vs. possessing ε2 or ε3 alleles) were performed. Sensitivity and subgroup analysis were also conducted to explore potential sources of heterogeneity. A one-by-one exclusion method was adopted by recalculating combined effect sized after removing one at the time each study. Subgroup analyses were conducted according to year of publication, study quality, age of participants at baseline, study design, length of follow-up, sample size, and type of dementia diagnosis. Small-study effects being the indicator of possible publication bias was examined quantitatively via Egger’s regression test as well as using graphical technique based on visual assessment of asymmetry patterns of funnel plots further adjusting for the number and outcomes of missing studies using trim-and-fill method. R version 4.3.0 (Development Core Team) was used for the statistical analysis. All tests were two-tailed and statistical significance was defined as *P* < 0.05.

## Results

A total of 1169 studies were deemed of potential interest for this systematic search. After removal of 130 duplicates and exclusion of 813 studies through title and abstract evaluation, the full-text from 226 studies were examined. An initial screening was applied based on the following reasons: lack of exposure (*n* = 85), no outcome of interest (*n* = 90), different study design (*n* = 2), only reported on biomarkers of consumption (*n* = 3), and conducted on younger population (*n* = 8). The resulting 38 studies were further examined for overlaps. After the final exclusion of 3 studies conducted on the same cohorts, a total of 35 studies [29–64] were included in the present meta-analysis (Supplementary Fig. 1).

### Study characteristics exploring fish consumption and cognitive outcomes

The main background characteristics of the studies included, and an overview of the main findings are reported in Table 1. A total of 25 had a prospective design, 8 were cross-sectional and 2 were case-control studies. Ten studies were conducted in Western countries, with 8 specifically involving Northern American cohorts and 13 conducted in European countries, while 13 including Eastern Asian countries. Most studies involved both sexes with just a few exceptions. Among the outcomes investigated through a variety of diagnostic and screening tools, 18 studies explored the relation between fish intake and cognitive decline 15 accounted for diagnosis of dementia, and 11 specifically investigated the risk of Alzheimer’s disease. In general (depending on the differential inclusion in specific analysis), the whole sample included a total of about 849,263 individuals, 8537 comprehensive cases of cognitive impairment/decline, 12,148 cases of dementia, and 5320 cases of Alzheimer’s disease. The quality of the studies scored over 5 for cross-sectional and over 7 for case-control and prospective studies, suggesting an overall good quality of the reports included in this meta-analysis (Supplementary Tables 4–6).

**Table 1:** General characteristics of included studies

Author, year	Country, cohort name (study design, follow-up)	Population (sex, mean age)	Total population, cases	Exposure, assessment method	Outcome	Outcome assessment method (modality of assessment)	Main findings	Adjustments
Broe, 1990	Australia (case-control)	Cases - new referrals to dementia clinics in Sydney aged 52–96 years (MF, 78.1)	T: 340, AD: 170	Fish, NA	AD	NINCDS-ADRDA, MMSE, Neurology of Aging Schedule, neuropsychological test battery (WMS, 7-word list learning test, CFST, COWAT, Porteus mazes, WAIS-Revised and NART) (clinical examination).	There was no association between fish consumption (never vs. any) and AD occurrence.	Matched for age and sex.
Kalmijn, 1997	The Netherlands, Zutphen Study (prospective, 3.0 y)	Dutch cohort of men aged 69–89 years (M, 75.0)	T: 476, CI: 153, CD: 51	Fish, cross-check dietary history method (2–4 weeks preceding the interview)	CI, CD	MMSE (self-reported screening).	High fish (> 20 g/d) consumption tended to be inversely associated with cognitive impairment (OR = 0.63, 95% Cl 0.33–1.21) and cognitive decline (OR = 0.45, 95% Cl 0.17–1.16).	Age, education, cigarette smoking, alcohol consumption, and energy intake.
Hebert, 2000	Canada, CSHA (prospective nested case-control)	Inhabitants of 10 Canadian provinces aged at least 65 years (MF, 76.2)	T: 907, VaD: 105	Shellfish, NA	VaD	DSM-III-R, DSM-IV-R, DSM-IV, NINDS-AIREN, CAMEX, NINCDS-ADRDA, ICD-10, 3MS, battery of neuropsychological tests (clinical examination).	Eating shellfish at least once per month was a significant protective factor of VaD (OR = 0.46, 95% CI: 0.22–0.88)	Age and region.
Barberger-Gateau, 2002	France, PAQUID (prospective, 7.0 y)	Inhabitants of 75 parishes in southwestern France aged 68 years and over (MF, NA)	T: 1416, D: 170, AD: 135	Fish and seafood, FFQ	D, AD	DSM-III-R criteria (clinical examination).	Eating fish or seafood at least once a week was associated with a significantly lower risk of dementia (age and sex adjusted HR = 0.66, 95% CI: 0.47–0.93). Additional controlling to education weakened the association and the result was on the boundary of significance (HR = 0.73, 95% CI: 0.52–1.03)	Age, sex, education.
Morris, 2003	US, CHAP (prospective, 3.9 y)	South-side Chicago Illinois community, residents 65 years and older (MF, 73.1)	T: 815, AD: 131	Fish, 139-item FFQ (modified Harvard version)	AD	CERAD, laboratory testing, MRI, NINCDS-ADRDA (clinical examination).	Fish consumption was inversely associated with the risk of incident AD. Consumption of at least 1 fish meal weekly was associated with 60% lower risk of AD than none or rare consumption. After restriction to persons with CVD conditions the association was even stronger.	Age, sex, race, education, total energy intake, APOE-e, race APOE-e interaction.
Huang, 2005	US, CHCS (prospective, 5.4 y)	Elderly American population (MF, 71.7)	T: 2233, D: 378, AD: 190	Fish, 99-item FFQ (modified version of the NCI)	D, AD	MMSE, 3MSE, DSS, BVRT, TICS, IQCoDE, DSM-IV, NINCDS-ADRDA, State of California ADDTC (clinical examination).	Consumption of fatty fish more than twice per week was associated with 28% and 41% lower risk of dementia, and AD respectively in comparison to those who ate fish less than once per month. Regarding APOE stratification, this effect was observed for non-carriers of ε4 allele.	Age at baseline, minority status, sex, presence of APOE ε4, energy, body mass index, region, education and income.
Barberger-Gateau, 2005	France, 3 C study (cross-sectional)	Bordeaux, Dijon, and Montpellier community dwellers aged 65 and over (MF, 74.3)	T: 9280, CI: NA	Fish, FFQ (1999–2000)	CI	MMSE (self-reported screening).	There was a negative association between the frequency of fish consumption and the risk of scoring below 24 on the MMSE.	Age, sex, education, and city.
Schaefer, 2006	US, FHS (prospective, 9.1 y)	Residents of the city of Framingham, Massachusetts, 55–88 years (MF, 76.0)	T: 899, D: 99, AD: 71	Fish, 126-item SFFQ	D, AD	Neuropsychological test battery, MMSE, a detailed case review by at least 2 neurologists, DSM-IV, NINCDS-ADRDA (clinical examination).	The adjusted RRs in subjects consuming fish more than twice a week compared with those consuming, at most, 2 servings of fish per week were 0.61 (95% CI: 0.28–1.33; *P* = 0.22) for all-cause dementia and 0.50 (95% CI: 0.20–1.27; *P* = 0.14) for Alzheimer disease.	Age, sex, apolipoprotein ε4 allele, plasma homocysteine concentration, and education level.
Almeida, 2006	Australia, Study in Australia (prospective, 4.8 y)	A community-representative cohort of Western Australialian men aged 75 years and over (M, 77.6)	T: 601, ICF: 106	Fish, NA (1996–1998)	CI	MMSE (self-reported screening).	Weekly consumption of fish was not associated with mental health	Crude.
Barberger-Gateau, 2007	France, 3 C study (prospective, 3.5 y)	Bordeaux, Dijon, and Montpellier community dwellers aged 65 and over (MF, NA)	T: 8085, D: 281, AD: 183	Fish, FFQ (1999–2000)	D, AD	Battery of neuropsychological tests, committee of expert neurologists, criteria of the DSM-IV and NINCDS-ADRDA (clinical examination).	Weekly consumption of fish was related to a reduced risk of AD (HR = 0.65, 95% CI: 0.43–0.99) as well as all cause dementia but only among ApoE ε4 noncarriers (HR = 0.60, 95% CI: 0.40–0.90).	Age, gender, education, city, income, and marital status, BMI, diabetes.
Vercambre, 2009	France, E3N (prospective, 13.0 y)	French epidemiological cohort of elderly women (F, 78.3)	T: 4809, CD: 598	Fish, 208-item FFQ (self-administered)	CD	DECO scale (self-reported screening).	Recent cognitive decline was associated with lower intake of fish	Age, education level, BMI, physical activity, energy intake, smoking status, supplement use, use of postmenopausal hormones, diabetes mellitus, hypertension, hypercholesterolaemia, history of CHD, stroke, cancer and depression.
Devore, 2009	Netherlands, Rotterdam Study (prospective, 9.6 y)	Residents of Ommoord (a district of Rotterdam) aged 55 y or older (MF, 67.7)	T: 5395, D: 465, AD: 365	Fish, 170-item SFFQ (1990–1993)	D, AD	MMSE, GMS, neurologist and neuropsychologist assessment, DSM-III-, NINCDS-ADRDA and NINDS-AIREN criteria (clinical examination).	Total and fatty fish intake was not associated with dementia risk (estimates comparing extreme categories were equal to HR = 0.95, 95% CI: 0.76–1.19 for total fish intake and HR = 0.98, 95% CI: 0.77–1.24 for fatty fish)	Age, sex, education, total energy intake, alcohol intake, smoking habits, BMI, high total cholesterol, hypertension at baseline, prevalent stroke, prevalent myocardial infarction, prevalent type 2 diabetes mellitus, dietary intake of vitamin E, and supplement use.
Roberts, 2010	US, MCSA (cross-sectional)	Olmsted County residents aged 70–89 years (MF, 80.1)	T: 1475, CD: 228	Fish, 128-item FFQ	MCI	CDR scale, DSM-IV (clinical examination).	Fish intake was not associated with mild cognitive impairment.	Age, sex, years of education, and total caloric intake, body mass index, ApoE ε4 allele, stroke, coronary heart disease, diabetes, and depression.
Lopez, 2011	US, RBS (cross-sectional)	Community dwelling men and women aged 67–100 years (MF, 80.2)	T: 266, D: 42, AD: 30	Fish, SFFQ (Harvard-Willett Diet Assessment Questionnaire)	D, AD	Neuropsychological tests (TMT (part B), Heaton VRT, Category Fluency, Buschke-Fuld SRT), neurological examination (NINCDS-ADRDA criteria) (clinical examination).	Eating at least one serving of fish per week was not associated with dementia (OR = 0.51, 95% CI: 0.20–1.32) and AD (OR = 0.55, 95% CI: 0.20–1.48)	Age, sex, apolipoprotein E status, education, and history of stroke.
Kesse-Guyot, 2011	France, SU.VI.MAX (prospective, 13.0 y)	France men and women who were older than 45 years (MF, 64.2)	T: 3294,	Fish, repeated 24-h dietary records	CI	MMSE, Verbal Memory, McNair’s Cognitive Difficulties Scale (self-reported screening).	Self-reported cognitive difficulties were borderline significantly less frequent among subjects with higher intakes of fish consumption (OR = 0.80, 95% CI = 0.63–1.01 for Q4 vs. Q1).	Gender, age, BMI, physical activity, tobacco status, education, energy intake, alcohol, baseline saturated fatty acids intake, n-6 fatty acids intake and fruit and vegetables consumption, baseline diabetes mellitus, hypercholesterolemia and hypertension and stroke and coronary heart diseases history; CES-D scale score.
Gao, 2011	China, SLAS (prospective, 1.5 y)	Chinese non-demented population aged at least 55 years (MF, 66.2)	T: 1475, CD: 226	Fish, FFQ	CI, CD	MMSE (self-reported screening).	No statistically significant association (OR = 1.02, 95% CI: 0.81–1.27) of fish consumption (3 or more times a week) with cognitive decline was found.	Age, gender, education, number of medical illness, the presence of vascular risk factors/diseases, smoking, alcohol drinking, depression, APOE status, nutritional status, level of leisure activity, baseline MMSE and length of follow-up.
Kim, 2013	US, WHS (prospective, 5.6 y)	Female health professionals in the United States who were aged 65 and older (F, 71.8)	T: 5988, cases: 10% the worst results	Fish and seafood, FFQ	CD	TICS (delayed recalls), MMSE, EBMT (immediate and delayed recalls), CERAD (category fluency) (self-reported screening).	At the first assessment compared with women who consumed < 1.0 serving/week of total seafood, those who consumed 1.1–2.0 servings/week had higher cognitive scores, but there were no differences by seafood consumption categories in overall change for a period of 4 years	Age, race, education, annual income, randomization assignment, alcohol intake, body mass index, exercise, current smoking, diabetes mellitus, hypertension, hypercholesterolemia, depression, total caloric intake, current hormone use, initial cognitive score, and time interval between the assessments.
Lee, 2017	Taiwan (cross-sectional)	A nationwide, population-based sample from 19 counties in Taiwan (MF, 75.7)	T: 10,432, D: 929	Fish, FFQ	MCI, D	NIA-AA criteria, CDR scale, TMSE, ADL, IADL, NIA-AA (self-reported screening).	Compared with rare consumption of fish, regular consumption of fish was associated with lower odds of dementia (OR = 0.62, 95% CI 0.41–0.94).	Age, gender, education, body mass index, dietary habit, exercise and comorbidity.
Bakre, 2018	China, Six-provinces study (cross-sectional)	Community from six Chinese provinces aged 60 years or older (MF, 72.2)	T: 6981, D: 326	Fish, FFQ	D	GMS, CSI-D, CERAD, AGECAT (self-reported screening).	Fish consumers had significantly reduced risk of dementia compared to non-consumers (OR = 0.73, 95% CI: 0.64–0.99). The dose–response relationship was not statistically significant.	Age, sex, province, urban/rural areas, education level, smoking status and stroke.
Tanaka, 2018	Italy, InCHIANTI (prospective, 10.1 y)	Residents of Greve in Chianti and and Bagno a Ripoli older than 65 years of age (MF, 75.4)	T: 832, CD: NA	Fish, FFQ (EPIC version)	CD	MMSE, DSM-IV, ADLs, IADLs, neuropsychological tests (paired words test, digit test from the WAIS, the Caltagirone drawings) (clinical examination).	Higher fish consumption (above median) was negatively associated with cognitive decline in model adjusted to age, sex and study site, but not in fully adjusted model	Age, sex, study site, chronic diseases, years of education, total energy intake, physical activity, BMI, ApoE4 carrier status, CRP, IL-6, plasma omega-3, plasma omega-6, plasma beta-carotene, plasma alpha-tocopherol.
Tsurumaki, 2019	Japan, Ohsaki study (prospective, 5.7 y)	Individuals aged ≥ 65 years living in Ohsaki City, Japan (F, 73.6)	T: 13,102, D: 1118	Fish, 39-item FFQ	D	Criteria of the LTCI system used in Japan (clinical examination).	Compared with subjects with the lowest fish intake (Q1), those with higher intake had reduced risk of dementia [HR = 0.90, 95% CI: 0.74–1.11) for Q2, HR = 0.85, 95% CI: 0.73–0.99 for Q3, and HR = 0.84, 95% CI: 0.71-0·997 for Q4; P for trend = 0·029].	Age, BMI, history of disease (stroke, hypertension, myocardial infarction, diabetes or hyperlipidaemia), education level, smoking, alcohol drinking, time spent walking, psychological distress score, cognitive function score, sleep duration, consumption volumes of green and yellow vegetables, fruits
Ngabirano, 2019	France, 3 C study (prospective, 9.8 y)	Volunteers aged 65 and over (MF, 76.9)	T: 5934, D: 662, AD: 466	Fish, FFQ	D, AD	Battery of neuropsychological tests, DSM-IV and NINCDS-ADRDA (clinical examination).	No significant association was found between the consumption of fish and the risk of dementia or AD.	Sex, inclusion centre, education level, income, marital status, APOE-ε4, tobacco, alcohol and physical activity, four other food categories (raw/cooked fruit/vegetable, meat) and caloric intake, BMI, diabetes, HBP, hypercholesterolemia and depression.
Chuang, 2019	Taiwan, NAHSIT (prospective, 11.0 y)	Noninstitutionalized people aged 65 years or older (MF, 73.2)	T: 1436, D: 260	Fish, FFQ (simplified version covering 16 items)	D	SPMSQ, MMSE, standard protocols for physician diagnosis (clinical examination).	Higher intake ( > = 4 vs. < 1 time/wk) of fish was associated with a lower risk of developing dementia, showing a long-term protective effect. No association between fish consumption and MCI was detected.	Age, sex, education, baseline cognition (SPSMQ), BMI, stroke history, DBP, inflammation status, and stroke occurrence during the study period (time-variated covariate).
Peeters, 2020	Cuba, 10/66 Dementia Research Group (prospective, 4.5 y)	Residents aged 65 years and over in urban and rural sites in Cuba (MF, 73.5)	T: 1846, D: 169	Fish, FFQ	D	GMS, CSI-D, VFT, object recognition and modified CERAD 10 word list, learning task with delayed recall, DSM-IV criteria (self-reported screening).	Rare fish consumption (vs. regular) was significantly associated with higher risk of dementia OR = 1.77, 95% CI: 1.06–2.95	Sex and education.
Keenan, 2020	US, AREDS (cross-sectional)	55–80 years participants with no AMD to unilateral AMD recruited at 11 US sites (MF, 68.7)	T: 3029, CI 3MS < 80: 119, CI CS:	Fish, 90-item semi-quantitative modified Block FFQ	CI	3MS (self-reported screening).	Fish intake was associated with higher cognitive function.	Age, sex, race, smoking, diabetes, hypertension, baseline depression score, total calorie intake, and (AREDS2 only) years from baseline.
Jiang, 2020	Singapore, SCHS (prospective, 20.0 y)	Middle-aged and older Chinese living in Singapore (MF, 53.5)	T: 16,948, CI: 2443	Fish and shellfish, 165-item semiquantitative FFQ (1993–1998)	CI	SM-MMSE (self-reported screening).	Higher fish/shellfish consumption appeared to be associated with lower risk of CI (OR = 0.87, 95% CI: 0.77–0.99 for Q3 vs. Q1 and OR = 0.89, 95% CI: 0.78–1.02 for Q4 vs. Q1). The inverse association was observed for fresh fish/shellfish whereas intake of preserved fish/selfish was associated with higher risk of CI (OR = 0.88, 95% CI 0.77-1.00 for extreme quartiles, P for trend = 0.03 for fresh fish/shellfish; OR = 1.19, 95% CI: 1.04–1.36, P for trend = 0.01 for preserved fish/shellfish).	Age at interview, gender, educational level, marital status, dialect groups, total energy intake, body mass index, physical activities, smoking status, alcohol consumption, sleep duration and self-reported physician diagnosis of diabetes, hypertension, heart disease, stroke, cancer, vegetables–fruit–soy dietary pattern, and the other two types of meat.
Filippini, 2020	Italy (case-control)	Cases - patients referred to the Cognitive Neurology Network of Modena province (MF, 65.0)	T: 138, EOD: 54, EO-AD: 30	Fish and seafood, 188-item EPIC-FFQ	EOD, EO-AD	Clinical criteria (NINCDS-ADRDA, DSM-IV, NINDS-AIREN) (clinical examination).	Overall fish and seafood consumption showed no association with EOD risk, however a U-shaped relation with preserved/tinned fish, and an inverse relation with other fish was found.	Sex, age, educational attainment, and energy intake.
Takeuchi, 2021	UK, UK Biobank (prospective, 10.0 y	Middle-aged population in the United Kingdom (MF, 56.5)	T: 341,591, D: 904	Fish, FFQ of 29 food groups (electronically self-reported)	D	ICD-9 and ICD-10, hospital inpatient records (HES (England), SMR (Scotland), PEDW) and death register data (NHS Digital (England), Wales ISD (Scotland) (clinical examination).	Moderately high total fish intake was significantly associated with decreased risk of subsequent onset of dementia.	Age, sex, neighbourhood-level socioeconomic status, education length, household income, current employment status, BMI, height, and race.
Nozaki, 2021	Japan, JPHC Study (prospective, 15.0 y	Japanese aged 45–64 at baseline (MF, 73.0)	T: 1127, D: 54	Fish and shellfish, 147-item FFQ (19 fish and shellfish items)	MCI, D	MMSE, WMS-R, logical memory I/II subtest, clock drawing test, J-ADNI criteria of dementia, DSM-IV, drawing test, and CDR Scale (clinical examination).	Higher fish consumption was significantly associated with reduced risks of dementia over non-dementia (MCI plus cognitively normal).	Age, sex, education, smoking status, alcohol frequency, physical activity, past history of cancer, myocardial infarction, diabetes mellitus, and depression.
Huang, 2021	China, CCSNSD (cross-sectional	Residents of Hebei, Zhejiang, Shaanxi, and Hunan provinces of China aged 55 years and older (MF, 68.4)	T: 4309, MCI: 605	Fish, 81-item SFFQ	MCI, D	MoCA [memory index score (MIS), executive index score (EIS), visuospatial index score (VIS), language index score (LIS), attention index score (AIS), and orientation index score (OIS)] (self-reported screening).	Consumers of higher amount of fish tended to have higher scores of global cognitive function and of particular domains, and to have lower odds of MCI, compared with the consumers of bottom level of fish.	Age, gender, residential area, education level, current employment, income level, physical activity, smoking, alcohol intake, sleep duration status, energy, disease history, obesity, and central obesity.
Zhang, 2021	China, CLHLS (prospective, 7.0 y)	Chinese community-dwelling older adults aged 65+ (MF, 77.8)	T: 3029, MSD: 685, SbCD: 723	Fish and seafood, face-to-face interviews by trained research staff	CD	MMSE (self-reported screening).	In APOE ε4 allele carriers daily fish intake was significantly associated with slower cognitive decline (OR = 0.43, 95% CI: 0.22–0.78). In non-carriers of APOE ε4 allele, the higher fish consumption did not affect cognitive performance.	Sex, age education, occupation before retirement, marital status, smoking, alcohol drinking, physical exercise, BMI, hypertension, diabetes, heart disease, and cerebrovascular disease.
Ylilauri, 2022	Finland, KIHD (prospective, 22.0 y)	Population-based sample of Finland men aged 42–60 years (M, 53.0)	T: 2497, D: 337, AD: 266	Fish, 4-day food records (using the picture book with 126 food items)	D, AD	The national health registers, ICD-8, ICD-9, ICD-10 (clinical examination).	No association between fish intake and dementia or AD risk. Higher fish consumption was associated with better verbal memory in all men and among APOE-ε4 carriers with better verbal fluency	Age, baseline examination year, energy intake, education years, pack-years of smoking, BMI, diabetes, leisure-time physical activity, history of coronary heart disease, use of lipid-lowering medication, intakes of alcohol, fiber, sum of fruits, berries and vegetables, dietary fat quality.
Xu, 2022	China, CLHLS (cross-sectional)	Chinese community-dwelling older adults aged 65+ (F, 83.2)	T: 7572, CI: 900	Fish, FFQ	CI	MMSE (self-reported screening).	In total sample no association between fish consumption and cognitive function was found. In participants over 80 years, higher daily intake of fish was associated with decreased risk of cognitive impairment.	Sex, age, years of schooling, rurality, BMI, physical exercise, marital status, sleep duration, alcohol consumption, smoking status, living status, diabetes, and hypertension.
Yeh, 2022	US, NHS and HPFS (prospective, 20.0 y)	Female registered nurses aged 30–55 y (NHS) and male health professionals aged 40–75 y (HPFS) residing in the United States (F, 48.0; M, 51.0)	M: 49,493, F: 27,842	Fish, SFFQs (7 times repeated from 1984 until 2006)	SCD	7-item (for females, NHS) or 6-item (for males, HPFS) questionnaire on memory and cognition changes (by e-mail or online questionnaires) (self-reported screening).	In females, decreasing dose-response trend of odds of SCD across fish intake categories was observed (OR = 0.83, 95% CI = 0.75, 0.90 for extreme categories comparison). No association between intake of fish and odds of SCD in males was found.	Age, total calorie intake, census tract income, education, husband’s education, race, smoking history, depression, physical activity level, BMI, intakes of alcohol, postmenopausal status, hormone replacement therapy, family history of dementia, missing indicator for SCD measurements, number of dietary assessments, multivitamin use, parity, dietary intakes of total vegetables, fruit, fruit juice, sweet/desserts, and sugar-sweetened beverages.
Huang, 2024	China, CLHLS (prospective, 8.1 y)	Chinese community-dwelling older adults aged 65+ (MF, 78.7)	T: 10,734, CI: 1220	Fish and seafood, FFQ of 10 food group items (face-to-face interviews)	CI	MMSE (self-reported screening).	Daily consumption of fish and aquatic products appeared to be negatively associated with risk of cognitive impairment, however the result did not reach significance (HR = 0.87, 95% CI: 0.72–1.05).	Age, sex, education, residence, marital status, co-residence, cohort, smoking status, exercise, leisure activity, engagement, BMI, dietary intakes of: fresh vegetables, fresh fruits, meat and poultry, eggs, legumes, preserved vegetables, sugar, tea, garlic.

Abbreviations:

F – females, M – males, MF – males and females, T – total sample

Names of examined cohorts: CSHA - Canadian Study of Health and Aging; PAQUID - Personnes Agées QUID; CHAP - Chicago Health and Aging Project; CHCS - Cardiovascular Health Cognition Study; 3 C study - Three-City study (in Bordeaux, Dijon, and Montpellier); FHS - Framingham Heart Study; E3N - Etude Epidemiologique de Femmes de la Mutuelle Generale de l’Education Nationale study; MCSA - Mayo Clinic Study of Aging; RBS - Rancho Bernardo Study, SU.VI.MAX study - Supplementation with Antioxidant Vitamins and Minerals study; SLAS - Singapore Longitudinal Aging Studies; WHS - Women’s Health Study; NAHSIT - Nutrition and Health Survey in Taiwan; 10/66 − 10/66 Dementia Research Group; AREDS - Age-Related Eye Disease Study; SCHS - Singapore Chinese Health Study; JPHC Study - Japan Public Health Center-based Prospective Study; CCSNSD - Community-based Cohort Study on Nervous System Diseases; CLHLS - Chinese Longitudinal Healthy Longevity Survey; KIHD - Kuopio Ischaemic Heart Disease Risk Factor Study; NHS - Nurses’ Health Study; EPIC - European Prospective Investigation in Cancer

Names of cognitive outcomes: CI - Cognitive impairment, MCI - Mild CI; CD - Cognitive decline, SCD - subjective CD, SbCD - substantial CD, MCD - moderate CD; D - dementia, VaD - Vascular dementia; AD - Alzheimer’s Disease; EOD Early-Onset Dementia, EO-AD - Early-Onset of AD; ICF - Impaired cognitive function

Names of tools used to assess cognitive function: MMSE - Mini-Mental State Examination, CMMSE - Chinese MMSE, CMMSE-r revised CMMSE, SM-MMSE -Singapore modified MMSE; 3MSE - Modified MMSE; FFQ - Food Frequency Questionnaire, SFFQ - semi-quantitative FFQ; TMT - Trail Making Test; VFL - Verbal Fluency (Test/Task); VRT - Visual Reproduction Test; SRT - Selective Reminding Test; ICD-8, ICD-9, ICD-10International Classification of Diseases (8, 9, 10); WMS, Wechsler Memory Scale, WMS-r - Wechsler Memory Scale Revised, WAIS - Wechsler Adult Intelligence Scale; CDR Scale - Clinical Dementia Rating Scale; J-ADNI - Japan Alzheimer’s Disease Neuroimaging Initiative; DSM Diagnostic and Statistical Manual of Mental Disorders (DSM-III, DSM-IV 3rd, 4th edition, DSM-III-R, DSM-IV-R - revised DSM-III, DSM-IV); MoCA - Montreal Cognitive Assessment; CS - Composite Score; CSI-D - Community Screening Instrument for Dementia; CERAD - Consortium to Establish a Registry for Alzheimer’s Disease; Telephone Interview for Cognitive Status (TICS-M - modified TICS); LTCI - Long term Care Insurance; SPMSQ - Short Portable Mental Status Questionnaires; ADDTC - Alzheimer’s Disease Diagnostic and Treatment Center; NCI - National Cancer Institute; NINCDS-ADRDA - National Institute of Neurological Disorders and Stroke and Alzheimer’s Disease and Related Disorders Association; NINDS-AIREN - National Institute of Neurological Disorders and Stroke and Association Internationale pour la Recherché et l´Enseignement en Neurosciences; ADDTC - Alzheimer’s Disease Diagnostic and Treatment Center; EBMT - East Boston Memory Test; BNMT - Boston Naming Test; DECO scale - Deterioration Cognitive Observee’ scale; GMS - Geriatric Mental State (GMS-A - organic level of GMS); DSS - Digit Symbol Substitution Test; BVRT - Benton Visual Retention Test; IQCoDE - Informant Questionnaire for Cognitive Decline in the Elderly; AGECAT - Automated Geriatric Examination for Computer Assisted Taxonomy; NIA-AA National Institute on Aging-Alzheimer’s Association; TMSE - Taiwanese Mental State Evaluation; NPI - Neuropsychiatric Inventory; AMDEX - Cambridge Examination for Mental Disorders of the Elderly; AMD - Age-related macular degeneration; HAMD - Hamilton Depression Scale; GDS - Geriatric Depression Scale; ADL - Activities of daily living; HES - Hospital Episode Statistics; NHS - National Health Service; SMR - Scottish Morbidity Records PEDW - Patient Episode Database for Wales; ISD - Information and Statistics Division; AgeCoDe - German Study on Aging, Cognition and Dementia in Primary Care Patients; RFT - randomised factorial trial; MRI - magnetic resonance imaging; LAMIC - low- and middle-income countries; BLS-D -Blessed - Dementia Scale; GVLT - Greek Verbal Learning Test; MCG - Medical College of Georgia Complex Figure Test; NART - National Adult Reading Test; CFST - Color Form Sorting Test; COWAT - Controlled Oral Word Association Test

### Comparison of the risk of cognitive disorders between extreme categories of fish intake

**Fig. 1:**
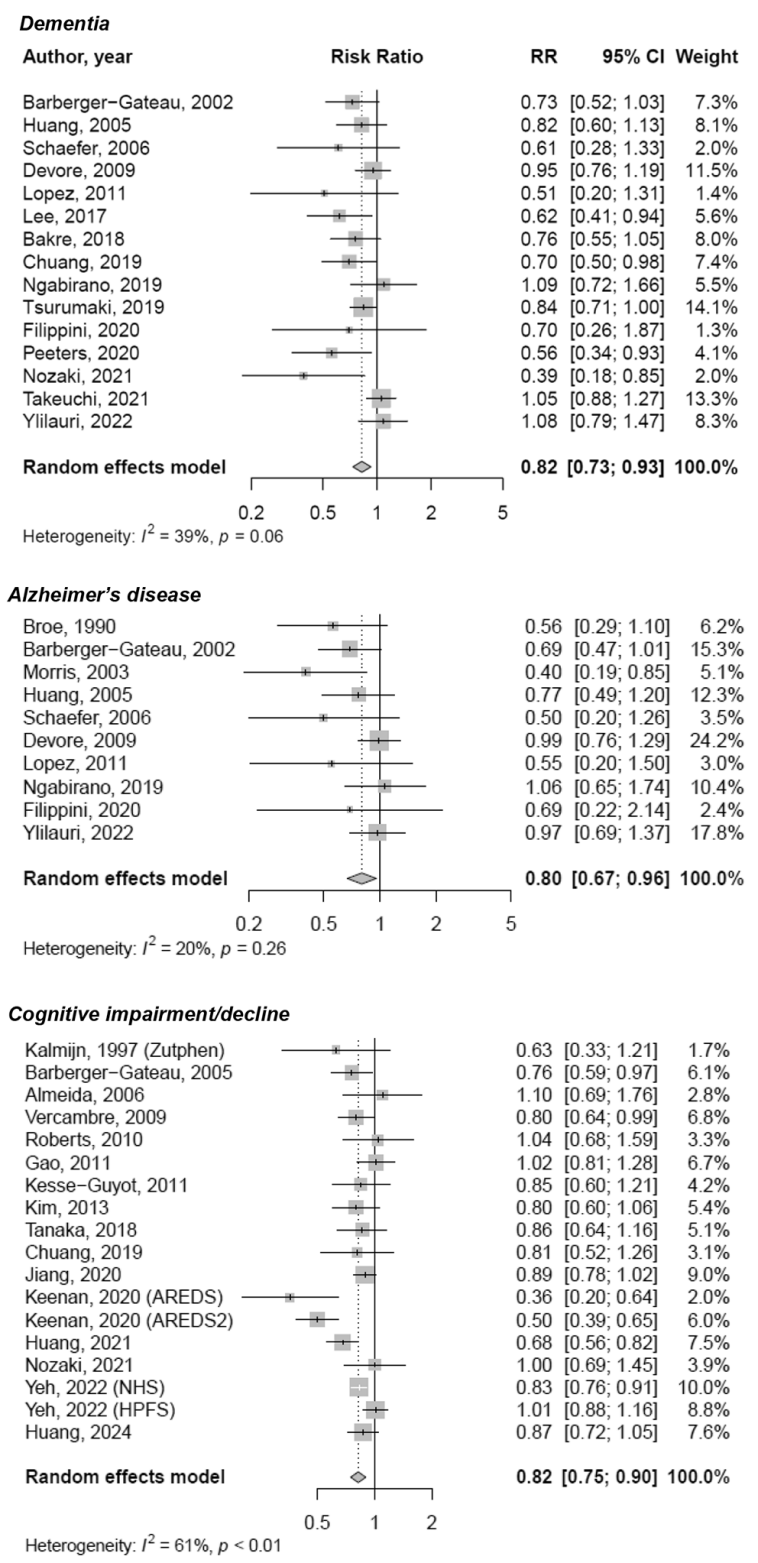
Meta-analysis of the risk of cognitive outcomes for the highest vs. the lowest fish consumption

The analysis of the association between fish consumption and dementia, Alzheimer’s disease, and cognitive impairment/decline was based on 15, 10 and 18 studies, respectively (Fig. 1 and Supplementary Table 7). Comparing with the lowest category of fish consumption, the highest consumption was related to 18%, 15% and 18% lower risk of each aforementioned outcome, respectively (RR = 0.82, 95% CI: 0.73–0.93 for dementia, RR = 0.80, 95% CI: 0.67–0.96 for Alzheimer’s disease, and RR = 0.82, 95% CI: 0.75–0.90 for cognitive impairment/decline; Fig. 1). The evidence of substantial heterogeneity was detected for cognitive impairment/decline (*I*^*2*^ = 61%, *P* < 0.001; Fig. 1). Exclusion of one study at the time did not considerably change any of the results (Supplementary Fig. 2); however, the analysis for cognitive impairment/decline risk resulted in a decrement of *I*^*2*^ statistic to 42% while still maintaining the similar estimate of size effect (RR = 0.76, 95% CI: 0.66–0.88) after exclusion of one study [52]. The inspection of funnel plots and the results of the Egger’s test revealed some evidence of asymmetry in all outcomes except for cognitive impairment/decline (Supplementary Fig. 3): trim-and-fill analysis adjusting for potential publication bias by complementing 5 and 4 missing studies in the case of dementia and Alzheimer’s disease, respectively, confirmed the previous findings (Supplementary Table 7, Supplementary Fig. 4). Subgroup analysis for each outcome showed substantially stable results, with some minor loss of significance in certain subgroups, such as results in European countries in the case of dementia and Alzheimer’s disease, analysis in studies with longer follow-up, and participants below 70 years (Table 2). Additional sub-group analyses focused on prospective studies only showed consistent results with only minor changes (i.e., no significant results in Asian countries, studies with longer follow-up, and larger samples for dementia) (Supplementary Table 8). Notably, most studies on cognitive decline relied on self-reported diagnosis through screening tools, while analyses on dementia and Alzheimer’s disease relied on clinical diagnosis, in both cases reporting stronger reduced risks with the most used diagnostic approach used (Supplementary Table 8).

**Table 2:** Subgroup analysis for risk of cognitive outcomes according to potential sources of heterogeneity the highest vs. the lowest fish consumption

Outcome/grouping variable	Subgroup category	*n*. studies	RR (95% CI)	*P*	I^2^	*P*_heter_	*P*_subgr_
*Dementia*							
Region	European countries	6	0.98 (0.88–1.10)	0.776	0.00	0.467	0.005
	Asian countries	5	0.74 (0.63–0.87)	0.000	24.02	0.261	
	Western countries	4	0.71 (0.55–0.90)	0.005	0.00	0.523	
Design	Prospective^&^	11	0.85 (0.75–0.97)	0.017	44.91	0.052	0.122
	CS/CC^&&^	4	0.69 (0.54–0.87)	0.002	0.00	0.800	
Year of publication	< 2018	6	0.80 (0.69–0.93)	0.005	6.19	0.377	0.649
	≥ 2018	9	0.84 (0.72–0.99)	0.040	51.64	0.035	
Follow-up, years	< 10^#^	11	0.81 (0.74–0.90)	0.000	0.00	0.470	0.779
	≥ 10^##^	4	0.85 (0.63–1.16)	0.311	69.76	0.019	
Sample size	< 5000	8	0.73 (0.59–0.91)	0.005	33.57	0.160	0.162
	≥ 5000	7	0.87 (0.77-1.00)	0.044	40.15	0.124	
Average age of participants, years	< 70	4	1.02 (0.89–1.16)	0.809	0.00	0.753	0.001
	≥ 70	11	0.76 (0.68–0.85)	0.000	6.70	0.380	
Modality assessment	Clinical examination	12	0.87 (0.77–0.98)	0.022	33.57	0.122	0.001
	Self-reported	3	0.67 (0.54–0.84)	0.001	0.00	0.546	
*Alzheimer’s Disease*							
Region	European countries	5	0.92 (0.77–1.09)	0.318	0.00	0.538	0.058
	Asian countries	1	0.56 (0.29–1.10)	0.090	0.00	1.000	
	Western countries	4	0.62 (0.44–0.86)	0.004	0.00	0.478	
Design	Prospective^&^	7	0.83 (0.67–1.01)	0.065	33.76	0.170	0.202
	CS/CC^&&^	3	0.58 (0.35–0.96)	0.033	0.00	0.946	
Year of publication	< 2018	7	0.71 (0.56–0.91)	0.006	32.07	0.183	0.089
	≥ 2018	3	0.98 (0.74–1.28)	0.868	0.00	0.791	
Follow-up, years	< 10^#^	9	0.76 (0.62–0.93)	0.009	22.19	0.246	0.233
	≥ 10^##^	1	0.97 (0.69–1.37)	0.862	0.00	1.000	
Sample size	< 5000	7	0.72 (0.56–0.92)	0.008	10.45	0.349	0.201
	≥ 5000	3	0.90 (0.70–1.15)	0.398	27.67	0.251	
Average age of participants, years	< 70	3	0.97 (0.79–1.19)	0.780	0.00	0.831	0.030
	≥ 70	7	0.70 (0.56–0.87)	0.001	2.66	0.405	
Modality assessment	Clinical examination	10	0.80 (0.67–0.96)	0.014	20.27	0.256	0.030
	Self-reported	-	-	-	-	-	
*Cognitive impairment/decline*							
Region	European countries	5	0.80 (0.70–0.91)	0.001	0.00	0.906	0.402
	Asian countries	7	0.87 (0.78–0.98)	0.023	40.14	0.124	
	Western countries	6	0.75 (0.60–0.94)	0.012	84.12	0.000	
Design	Prospective^&^	13	0.85 (0.77–0.94)	0.001	57.91	0.005	0.180
	CS/CC^&&^	5	0.72 (0.57–0.90)	0.005	56.24	0.058	
Year of publication	< 2018	8	0.86 (0.78–0.96)	0.006	0.00	0.480	0.303
	≥ 2018	10	0.79 (0.69–0.90)	0.000	75.58	0.000	
Follow-up, years	< 10^#^	11	0.76 (0.65–0.90)	0.001	68.15	0.001	0.112
	≥ 10^##^	7	0.88 (0.82–0.94)	0.000	6.72	0.377	
Sample size	< 5000	12	0.78 (0.67–0.92)	0.003	66.19	0.001	0.239
	≥ 5000	6	0.87 (0.81–0.94)	0.000	26.69	0.234	
Average age of participants, years	< 70	7	0.84 (0.74–0.96)	0.011	73.20	0.001	0.633
	≥ 70	11	0.80 (0.71–0.91)	0.001	48.07	0.037	
Modality assessment	Clinical examination	4	0.92 (0.76–1.10)	0.350	0.00	0.793	0.633
	Self-reported	14	0.80 (0.72–0.89)	0.000	68.87	0.000	

### Comparison of the risk of cognitive disorders dependent on the amount of fish consumption

**Fig. 2:**
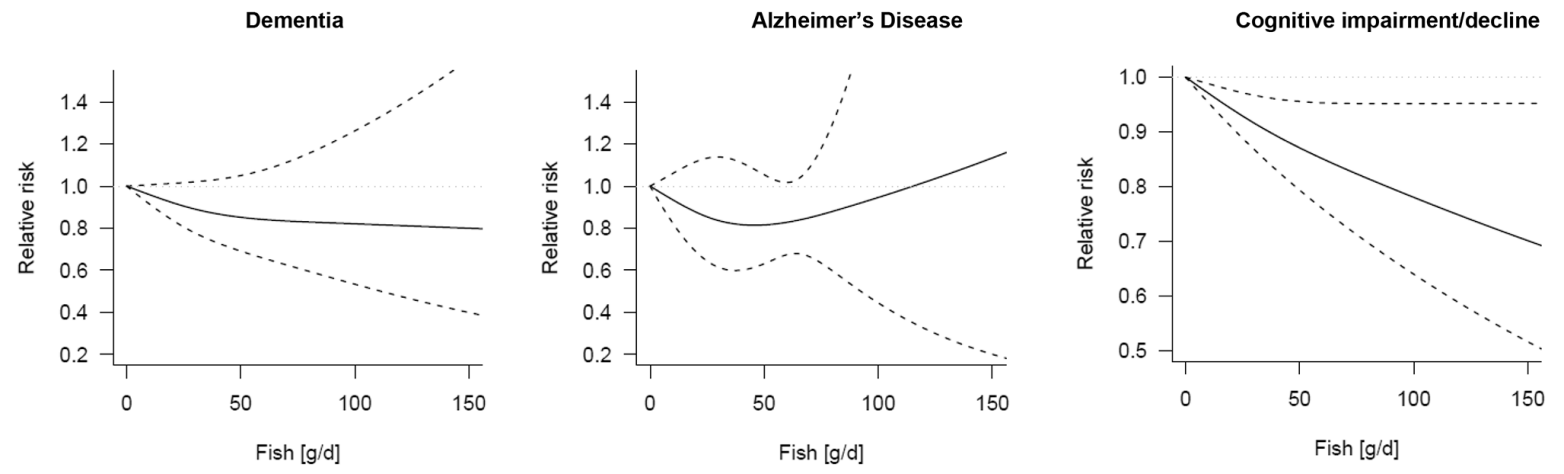
Graphical representation of dose-response meta-analysis of the risk of cognitive outcomes for various servings of fish intake

The dose-response analysis using restricted cubic splines is graphically presented in Fig. 2 and RRs are reported in Table 3. A significant decreased risk of cognitive impairment/decline across higher levels of fish intake up to 30% for 150 g/d was found (RR = 0.70, 95% CI: 0.52–0.95), although with large confidence intervals and evidence of significant heterogeneity (*I²* >90%, *P* < 0.001). No significant findings were found for dementia and Alzheimer’s disease, although a decreased risk of the latter for up to 50 g/d of fish was reported (Fig. 2**and** Table 3).

**Table 3:** Dose-response meta-analysis of the risk of cognitive outcomes for various servings of fish intake

		RR (95% CI) for different levels of daily fish intake					Heterogeneity	
Outcome	*n*. studies	0 g/d	50 g/d	100 g/d	150 g/d	*P*_nonlin_	I^2^	*P*_heter_
*All studies*								
Dementia	11	1 (REF)	0.85 (0.69–1.05)	0.82 (0.53–1.26)	0.80 (0.40–1.60)	0.257	61.9	< 0.001
Alzheimer’s Disease	6	1 (REF)	0.82 (0.63–1.05)	0.95 (0.44–2.01)	1.13 (0.20–6.42)	0.523	7.6	0.371
Cognitive impairment/decline	8	1 (REF)	0.87 (0.79–0.96)	0.78 (0.64–0.95)	0.70 (0.52–0.95)	0.010	98.1	< 0.001

### Fish intake and cognitive outcomes by APOE genotype strata analysis

A limited number of studies presented results by APOE genotype strata (up to 3 studies, dependent on the endpoint) and results were rather contrasting (Supplementary Fig. 5 and Supplementary Table 9). Results from pooled analyses for dementia resulted in null findings although with a trend toward decreased risk in APO ε2 or ε3 allele carriers (RR = 0.77, 95% CI: 0.58–1.03). However, the only study specifically conducted on cognitive impairment/decline showed a decreased risk for higher fish consumers among APOE ε4 allele carriers (RR = 0.18, 95% CI: 0.05–0.63). No significant associations were found between fish intake and Alzheimer’s disease risk by APOE genotype strata.

## Discussion

The aim of this study was to explore the relation between fish consumption and the risk of a variety of cognitive outcomes in observational studies. The main results of the meta-analysis showed that higher fish consumption was associated with lower risk of cognitive impairment/decline, dementia, and Alzheimer’s disease, although a clear dose-response relation could only be observed for the former. A certain degree of heterogeneity could only be partially explained by some variables (i.e., age groups), while differences by genetic background may in fact play a role, yet only limitedly investigated, with not enough studies to effectively draft conclusions on this matter.

Aside from a para-physiological decline in cognitive abilities associated with the growing age, pathological cognitive impairment may depend on a variety of changes in the older brain both determined by genetic and environmental stimuli [65]. Alteration of brain structure, neurotransmission, vascular irroration, and deposit of abnormal proteins (i.e., beta amyloid) are the most common pathological processes determining an alteration of cognitive abilities in older individuals [66–68]. Fish is rich in omega-3 PUFA, which have been widely demonstrated to exert a variety of actions in the human brain, including modulating the immune response to insulting stimuli and eventually counteracting neuroinflammation by serving as precursors of pro-resolving mediators, affecting nitric oxide synthesis, decreasing reactive oxygen species (ROS), and more in general improving endothelial dysfunction characterizing certain types of dementia [69–71]. Omega-3 PUFA also play an important role in maintaining structural function of the brain, preserving the integrity of the blood-brain barrier, counteract brain atrophy, promoting neurogenesis and increased volume of certain brain area deputed to cognitive functions, the hippocampus [72–74]. Although much evidence for such mechanisms is often supported by only preclinical models, current findings match the rationale behind the results from most observational studies conducted so far on fish and cognitive outcomes.

Aside from omega-3 PUFA, recent research has focused on other components of fish that may result in effects on the human brain. Oligopeptides found in fish have been shown to potentially exert neuroprotective effects by serving as precursors of biologically active agents that may counteract some processes occurring in the brain promoting cognitive decline [75]. Some of the mechanisms potentially playing a role against neurodegenerative diseases reported to be exerted by bioactive peptides from seafood include modulation of inflammatory pathways and pro-survival and neurotrophic gene expression, improvement of cell viability, inhibition of acetylcholinesterase and endothelial nitric oxide synthase, and reduction of intracellular antioxidant enzymes depletion [76]. Moreover, inhibitory effects on the beta-secretase enzyme involved in the generation of amyloid-beta peptides that aggregate in the brain of Alzheimer’s patients have also been reported from marine-derived peptides [77]. Although most evidence is yet based on preclinical studies, there is much interest in further investigating the efficacy of such compounds in human in vivo trials. Certain limitations should be considered when exploring their actual capacity to exert effects on human health, including the resistance to digestion operated by proteases and peptidases occurring all over the gastrointestinal tract and the capacity to cross the blood-brain barrier [78]. Nonetheless, the aforementioned mechanisms could support the hypothesis that neuroprotective peptides from fish could play a role against cognitive impairment.

Fish is also a rich source of vitamins and minerals that can play, to a certain extent, a role in brain health [79]. A large variety of minerals, such as iron, magnesium, zinc, phosphorus, and selenium, as well as vitamins, such as group B and D vitamins, are generally well represented in seafood. While there is not much evidence of meaningful effects of supplementation on cognitive decline or dementia [80], all the aforementioned micronutrients are known to play important physiological actions in brain cells, including maintenance of a functional neuroglia, synthesis of precursors of neurotransmitters and control of intracellular calcium release, both important for synaptic excitability and neurotransmission [81]. Nutritional deficiencies lead to documented neurological malfunctioning possibly due to failure of defense mechanisms (i.e., against oxidative stress and inflammation) or age-related frailty, including fatigue and decrease in cognitive performance [82]. Although it is unclear whether the vitamin and mineral content in fish may play a substantial role in preventive dementia and Alzheimer’s disease, they are most likely to affect cognitive abilities and long-term exposure or, on the contrary, chronic deficiency may in fact be an important factor for the maintenance of a healthy brain and decrease the risk of neurodegenerative conditions [83].

The findings on the associating between fish and cognitive outcomes may display a certain degree of heterogeneity across studies because of some variables that should be taken into account when exploring such topic. First, the positive effects of intake of omega-3 toward the central nervous system has been demonstrated to be valid in individuals with cognitive decline or dementia, although the impact on the basic pathological lesion (i.e., amyloid deposition) and more advanced stages of dementia is still unclear [84, 85]. Other factors to be considered to interpret heterogeneity of results include the potential discrepancy between omega-3 PUFA dietary consumption, plasma concentrations, and brain membrane composition, which may be eventually influenced by age or genetic factors [86]. In fact, older individuals may have been suggested to exhibit higher omega-3 PUFA plasma concentrations and yet lower content in their brains [87, 88], leading to a higher susceptibility to potential deficiency and, consequently, stronger effect following exposure. This hypothesis is in line with the results of the present study, being the retrieved association reported especially in individuals older than 70 years old. Among genetic factors, APOE variants (a lipid transporter within the brain) and genes encoding enzymes involved in the leukotriene synthesis, has been shown to interact, albeit with contrasting results, with dietary PUFA intake and their related health effects [89]. Such strata analysis has also been performed in the present meta-analysis and the results pointed to a potential weaker association between fish consumption and APOE ε4 allele, a genetic marker associated with disturbed omega-3 PUFA metabolism leading to lower plasma concentrations than in non-carriers [90, 91], in which significant associations with lower risk of dementia and cognitive impairment were found. Other potential sources of heterogeneity may depend on the type of outcome investigated as well as the type of diagnosis (evaluation through screening tools vs. clinical assessment). For both variables, considering the results reported in this study, we may hypothesize that the risk reduction may occur when considering age-related cognitive decline or generic cognitive deficits and disorders, not just yet developed into well-identified clinical conditions, such as certain types of dementia, including Alzheimer’s disease. Eventually, other factors (i.e., genetic and environmental) may concur to the development of specific neurocognitive disfunctions and diet alone may not be sufficient to actually significantly reduce the risk of their insurgence (at least not observed for fish with the models currently used).

Other limitations potentially determining the heterogeneity of the results (as well as affecting the strength of evidence) include technical and methodological features from the original studies included in the meta-analysis. First, most studies used self-reported dietary information to assess fish consumption, which may be subject to recall bias and social desirability bias. Second, although the quality of the included studies was high, the original study design cannot detect any causal inference, but only associations with risk. Moreover, the adjustment for several potential confounding factors do not guarantee the presence of unmeasured variables potentially playing a role in brain health (i.e., overall diet quality). Ultimately, fish consumption is relatively easy to be estimated and would allow to consider not only the role of omega-3 PUFA but also other components potentially important to exert putative effects on human brain: nonetheless, investigating specific markers in blood or brain would be a further necessary step to increase precision of measurements and inference, ultimately potentially reducing the heterogeneity of findings. Finally, concerning the outcomes, the use of several different tools across the studies may limit the univocity and the consistency of the endpoints investigated.

In conclusion, the present study showed that higher fish intake may be associated with better cognitive status in older individuals. Whether fish consumption may actually decrease the risk of dementia and Alzheimer’s disease is still to be confirmed, but current results are promising. The observed trends of risk estimates suggest a lower risk of disease with increasing consumption of fish starting 50 g per day, while findings for higher intakes are more heterogeneous. The existing mechanistic evidence providing a sound rationale in support of such findings and the consistency of results foster the inclusion of fish in a healthy, balanced dietary pattern. While fish consumption may naturally occur in more coastal areas or, more in general, countries with historical and cultural habits characterized by its inclusion in traditional dietary patterns (i.e., the Mediterranean diet), it is important to consider the importance of food availability and affordability globally. Although evidence from the scientific literature support the mechanical role of omega-3 PUFA in improving certain brain structure, further studies are needed to shed the doubts concerning the actual role of fish intake in more pathophysiological complex conditions, to estimate whether the beneficial effects are in fact exerted by its content in omega-3 or rather other compounds (such as, peptides), and to understand the extent of efficacy also in relation to age and genetic factors. Although interesting and giving the chance for speculation, the findings of this study are yet preliminary and need additional proof to further investigate the role of unmeasured factors, including mechanisms (such as, transportation, membrane incorporation, etc.) related to personalized inter-individual response to omega-3 PUFA or other nutritional compounds retrieved in fish. Also, a more precise clinical characterization of cognitive disorders could help to reduce the heterogeneity of findings and identifying potential specific conditions particularly sensible to the beneficial effects of inclusion of fish in a healthy diet.

### Electronic supplementary material

Below is the link to the electronic supplementary material.

Supplementary Material 1

## Data Availability

All data supporting the findings of this study are available from the corresponding author upon reasonable request.
