# Supplementary material for: Fish consumption, cognitive impairment and dementia: an updated dose-response meta-analysis of observational studies
Source: Aging Clin Exp Res. 2024 Aug 20;36(1):171. doi: 10.1007/s40520-024-02823-6 (PMC11335789; doi:10.1007/s40520-024-02823-6)

Supplementary Table 1. The Meta-analysis of Observational Studies in Epidemiology (MOOSE) guidelines.

| **Section/topic** | **#** | **Checklist item** | **Reported on page #** |
| --- | --- | --- | --- |
| **TITLE** | | |  |
| Title | 1 | Identify the report as a systematic review, meta-analysis, or both. | 1 |
| **ABSTRACT** | | |  |
| Structured summary | 2 | Provide a structured summary including, as applicable: background; objectives; data sources; study eligibility criteria, participants, and interventions; study appraisal and synthesis methods; results; limitations; conclusions and implications of key findings; systematic review registration number. | 3 |
| **INTRODUCTION** | | |  |
| Rationale | 3 | Describe the rationale for the review in the context of what is already known. | 3,4 |
| Objectives | 4 | Provide an explicit statement of questions being addressed with reference to participants, interventions, comparisons, outcomes, and study design (PICOS). | 3,4 |
| **METHODS** | | |  |
| Protocol and registration | 5 | Indicate if a review protocol exists, if and where it can be accessed (e.g., Web address), and, if available, provide registration information including registration number. | 4 |
| Eligibility criteria | 6 | Specify study characteristics (e.g., PICOS, length of follow-up) and report characteristics (e.g., years considered, language, publication status) used as criteria for eligibility, giving rationale. | 5 |
| Information sources | 7 | Describe all information sources (e.g., databases with dates of coverage, contact with study authors to identify additional studies) in the search and date last searched. | 5 |
| Search | 8 | Present full electronic search strategy for at least one database, including any limits used, such that it could be repeated. | Supp matt |
| Study selection | 9 | State the process for selecting studies (i.e., screening, eligibility, included in systematic review, and, if applicable, included in the meta-analysis). | 5 |
| Data collection process | 10 | Describe method of data extraction from reports (e.g., piloted forms, independently, in duplicate) and any processes for obtaining and confirming data from investigators. | 5 |
| Data items | 11 | List and define all variables for which data were sought (e.g., PICOS, funding sources) and any assumptions and simplifications made. | 5 |
| Risk of bias in individual studies | 12 | Describe methods used for assessing risk of bias of individual studies (including specification of whether this was done at the study or outcome level), and how this information is to be used in any data synthesis. | 5 |
| Summary measures | 13 | State the principal summary measures (e.g., risk ratio, difference in means). | 6 |
| Synthesis of results | 14 | Describe the methods of handling data and combining results of studies, if done, including measures of consistency (e.g., I^2^) for each meta-analysis. | 6 |
| Risk of bias across studies | 15 | Specify any assessment of risk of bias that may affect the cumulative evidence (e.g., publication bias, selective reporting within studies). | 6 |
| Additional analyses | 16 | Describe methods of additional analyses (e.g., sensitivity or subgroup analyses, meta-regression), if done, indicating which were pre-specified. | 6 |
| **RESULTS** | | |  |
| Study selection | 17 | Give numbers of studies screened, assessed for eligibility, and included in the review, with reasons for exclusions at each stage, ideally with a flow diagram. | 7,8 |
| Study characteristics | 18 | For each study, present characteristics for which data were extracted (e.g., study size, PICOS, follow-up period) and provide the citations. | 8 |
| Risk of bias within studies | 19 | Present data on risk of bias of each study and, if available, any outcome level assessment (see item 12). | 8 |
| Results of individual studies | 20 | For all outcomes considered (benefits or harms), present, for each study: (a) simple summary data for each intervention group (b) effect estimates and confidence intervals, ideally with a forest plot. | Table 1 |
| Synthesis of results | 21 | Present results of each meta-analysis done, including confidence intervals and measures of consistency. | 8,9 |
| Risk of bias across studies | 22 | Present results of any assessment of risk of bias across studies (see Item 15). | 8,9 |
| Additional analysis | 23 | Give results of additional analyses, if done (e.g., sensitivity or subgroup analyses, meta-regression [see Item 16]). | 8,9 |
| **DISCUSSION** | | |  |
| Summary of evidence | 24 | Summarize the main findings including the strength of evidence for each main outcome; consider their relevance to key groups (e.g., healthcare providers, users, and policy makers). | 10-12 |
| Limitations | 25 | Discuss limitations at study and outcome level (e.g., risk of bias), and at review-level (e.g., incomplete retrieval of identified research, reporting bias). | 12,13 |
| Conclusions | 26 | Provide a general interpretation of the results in the context of other evidence, and implications for future research. | 13 |
| **FUNDING** | | |  |
| Funding | 27 | Describe sources of funding for the systematic review and other support (e.g., supply of data); role of funders for the systematic review. | 13 |

Supplementary Table 2. Search strategy.

| Pubmed | |
| --- | --- |
| 1 | ("fishes"[MeSH Terms] OR "fishes"[All Fields] OR "fish"[All Fields] OR "seafood"[MeSH Terms] OR "seafood"[All Fields] OR "seafoods"[All Fields] OR "shellfish"[MeSH Terms] OR "shellfish"[All Fields] OR "shellfishes"[All Fields]) |
| 2 | (“cognitive dysfunction"[MeSH Terms] OR "cognition"[MeSH Terms] OR "cognition"[All Fields] OR "cognitions"[All Fields] OR "cognitive"[All Fields] OR "cognitively"[All Fields] OR "cognitives"[All Fields] OR "memories"[All Fields] OR "memory"[MeSH Terms] OR "memory"[All Fields] OR "memory s"[All Fields] OR "dementia"[MeSH Terms] OR "dementia"[All Fields] OR "dementias"[All Fields] OR "dementia s"[All Fields] OR "alzheime s"[All Fields] OR "alzheimer disease"[MeSH Terms] OR ("alzheimer"[All Fields] AND "disease"[All Fields]) OR "alzheimer disease"[All Fields] OR "alzheimer"[All Fields] OR "alzheimers"[All Fields] OR "alzheimer s"[All Fields] OR "alzheimers s"[All Fields] ​​OR "neurodegenerative"[All Fields] OR "neurodegeneration"[All Fields] OR "Neurodegenerative Diseases"[MeSH Terms] OR "intellectual impairment"[All Fields] OR "intellectual dysfunction"[All Fields]) |
| 3 | ("prospective"[All Fields] OR "prospectively"[All Fields] OR "longitudinal"[All Fields] OR "longitudinally"[All Fields] OR "cohort"[All Fields] OR "cohort s"[All Fields] OR "cohorte"[All Fields] OR "cohorts"[All Fields] OR "observational"[All Fields] OR "follow-up"[All Fields] OR "nested"[All Fields] OR "case-control"[All Fields] OR "cross-sectional"[All Fields] OR "population-based"[All Fields] OR "Cohort Studies"[MeSH Terms] OR "Longitudinal Studies"[MeSH Terms] OR "Prospective Studies"[MeSH Terms] OR "Cross-Sectional Studies"[MeSH Terms] OR "Case-Control Studies"[MeSH Terms] OR "Epidemiologic Studies"[MeSH Terms] OR "Longitudinal Studies"[MeSH Terms] OR "Observational Study"[Publication Type]) |
|  | 1 AND 2 AND 3 |
| Scopus | |
|  | TITLE-ABS-KEY ( ( fish OR seafood OR shellfish ) AND ( cognitive OR memory OR dementia OR alzheimer's OR alzheimer ) AND ( prospective OR prospectively OR longitudinal OR cohort OR cohorts OR observational OR follow-up OR nested OR case-control OR cross-sectional ) ) |

Supplementary Table 3. PICOS criteria.

| **Parameter** | **Description** |
| --- | --- |
| (P) Population | Older adults |
| (I) Intervention/Exposure | Habitual fish, seafood or shellfish consumption |
| (C) Comparison | Different categories of habitual fish, seafood or shellfish consumption |
| (O) Outcome | Cognitive disorders |
| (S) Study design | Observational studies with a comparison group (cohort studies, cross-sectional studies, case-control studies) |

Supplementary Table 4. Assessment of study quality according to the Newcastle-Ottawa Quality Assessment Scale for cross-sectional studies.

|  | SELECTION | | | COMPARABILITY | EXPOSURE | | TOTAL SCORE |
| --- | --- | --- | --- | --- | --- | --- | --- |
|  | Representativeness of the exposed cohort | Non-respondents | Ascertainment of exposure |  | Assessment of outcome | Statistical test |  |
| Xu 2022 | * | - | * | ** | * | * | 6 |
| Huang 2021 | * | - | * | ** | * | * | 6 |
| Keenan 2020 | - | - | * | ** | * | * | 5 |
| Bakre 2018 | * | - | * | ** | * | * | 6 |
| Lee 2017 | * | - | * | ** | * | * | 6 |
| Roberts 2010 | - | - | * | ** | * | * | 5 |
| Barberger-Gateau 2005 | * | - | * | ** | * | * | 6 |

Supplementary Table 5. Assessment of study quality according to the Newcastle-Ottawa Quality Assessment Scale for case-control studies.

|  | SELECTION | | | | COMPARABILITY | EXPOSURE | | | TOTAL SCORE |
| --- | --- | --- | --- | --- | --- | --- | --- | --- | --- |
|  | Case definition adequate | Representativeness of the cases | Selection of controls | Definition of controls |  | Assessment of exposure | Same methods for cases and controls | Non-response rate |  |
| Filippini 2020 | * | * | * | * | ** | * | * | * | 9 |
| Broe 1990 | * | * | * | * | ** | * | * | * | 9 |

Supplementary Table 6. Assessment of study quality according to the Newcastle-Ottawa Quality Assessment Scale for prospective studies.

|  | SELECTION | | | | COMPARABILITY | EXPOSURE | | | TOTAL SCORE |
| --- | --- | --- | --- | --- | --- | --- | --- | --- | --- |
|  | Representativeness of the exposed cohort | Selection of the non-exposed cohort | Ascertainment of exposure | Demonstration that outcome of interest was not present at start of study |  | Assessment of outcome | Was follow-up long enough for outcomes to occur | Adequacy of follow up of cohorts |  |
| Huang 2024 | * | * | * | * | ** | * | * | * | 9 |
| Ylilauri 2022 | * | * | * | * | ** | * | * | * | 9 |
| Yeh 2022 | * | * | * | * | ** | * | * | * | 9 |
| Dobreva 2022 | * | * | * | * | ** | * | * | * | 9 |
| Takeuchi 2021 | * | * | * | * | ** | * | * | * | 9 |
| Nozaki 2021 | * | * | * | * | ** | * | * | * | 9 |
| Peeters 2020 | * | * | * | * | ** | * | * | * | 9 |
| Jiang 2020 | * | * | * | * | ** | * | * | * | 9 |
| Tsurumaki 2019 | * | * | * | * | ** | * | * | * | 9 |
| Ngabirano 2019 | * | * | * | * | ** | * | * | * | 9 |
| Kim 2013 | * | * | * | - | ** | * | * | * | 8 |
| Lopez 2011 | * | * | * | * | ** | * | * | * | 9 |
| Kesse-Guyot 2011 | * | * | * | * | ** | * | * | * | 9 |
| Vercambre 2009 | * | * | * | * | ** | * | * | * | 9 |
| Devore 2009 | * | * | * | * | ** | * | * | * | 9 |
| Barberger-Gateau 2007 | * | * | * | * | ** | * | * | * | 9 |
| Schaefer 2006 | * | * | * | * | ** | * | * | * | 9 |
| Huang 2005 | * | * | * | * | ** | * | * | * | 9 |
| Morris 2003 | * | * | * | * | ** | * | * | * | 9 |
| Kalmijn a 1997 | * | * | * | * | ** | * | * | * | 9 |
| Kalmijn b 1997 | * | * | * | * | ** | * | * | * | 9 |
| Barberger-Gateau 2002 | * | * | * | * | ** | * | * | * | 9 |
| Gao 2011 | * | * | * | * | ** | * | * | * | 9 |
| Roberts 2010 | * | * | * | * | ** | * | * | * | 9 |
| Samieri 2008 | * | * | * | * | ** | * | * | * | 9 |
| Hebert 2000 | * | * | * | * | * | * | * | - | 7 |
| Larrieu 2004 | * | * | * | * | ** | * | * | * | 9 |
| Chuang 2019 | * | * | * | * | ** | * | * | * | 9 |
| Tanaka 2018 | * | * | * | * | ** | * | * | - | 8 |
|  |  |  |  |  |  |  |  |  |  |

Supplementary Table 7. Comparison of the risk of specific cognitive outcomes between individuals reporting the highest *vs*. the lowest fish intake.

| Outcome | n. studies | RR (95% CI) | *P* | *I^2^* | *P_heter_* |
| --- | --- | --- | --- | --- | --- |
| *The main analysis* |  |  |  |  |  |
| Dementia | 15 | 0.82 (0.73-0.93) | 0.001 | 38.7 | 0.063 |
| Alzheimer's Disease | 10 | 0.80 (0.67-0.96) | 0.014 | 20.3 | 0.256 |
| Cognitive impairment/decline | 18 | 0.82 (0.75-0.90) | <0.001 | 61.1 | <0.001 |
| *Trim-and-fill analysis* |  |  |  |  |  |
| Dementia | 20# | 0.88 (0.78-1.00) | 0.050 | 47.8 | 0.009 |
| Alzheimer's Disease | 14# | 0.89 (0.73-1.08) | 0.228 | 38.6 | 0.070 |
| Cognitive impairment/decline | 18# | 0.82 (0.75-0.90) | <0.001 | 61.1 | <0.001 |
| ^#^number of studies complemented based on trim-and-fill method to adjust for bias in meta-analysis | | | | | |

Supplementary Table 8. Subgroup analysis for risk of cognitive outcomes according to potential sources of heterogeneity the highest *vs*. the lowest fish consumption in prospective studies.

| Outcome/grouping variable | Subgroup category | n. studies | RR (95% CI) | *P* | *I^2^* | *P_heter_* | *P_subgr_* |
| --- | --- | --- | --- | --- | --- | --- | --- |
| *Dementia* |  |  |  |  |  |  |  |
| Region | European countries | 3 | 0.72 (0.56-0.93) | 0.012 | 0.0 | 0.415 | 0.025 |
|  | Asian countries | 5 | 0.99 (0.88-1.11) | 0.831 | 3.2 | 0.388 |  |
|  | Western countries | 3 | 0.72 (0.54-0.97) | 0.029 | 51.4 | 0.128 |  |
| Design | Prospective^&^ |  |  |  |  |  |  |
|  | CS/CC^&&^ |  |  |  |  |  |  |
| Year of publication | <2018 | 4 | 0.85 (0.73-1.00) | 0.044 | 0.0 | 0.484 | 0.964 |
|  | ≥2018 | 7 | 0.85 (0.71-1.03) | 0.108 | 61.0 | 0.018 |  |
| Follow-up, years | <10^#^ | 7 | 0.84 (0.75-0.95) | 0.004 | 6.0 | 0.381 | 0.937 |
|  | ≥10^##^ | 4 | 0.85 (0.63-1.16) | 0.311 | 69.8 | 0.019 |  |
| Sample size | <5000 | 6 | 0.73 (0.57-0.94) | 0.016 | 48.6 | 0.083 | 0.111 |
|  | ≥5000 | 5 | 0.92 (0.81-1.05) | 0.219 | 29.1 | 0.228 |  |
| Average age of participants, years | <70 | 3 | 1.02 (0.90-1.16) | 0.736 | 0.0 | 0.726 | 0.004 |
|  | ≥70 | 8 | 0.77 (0.67-0.89) | 0.000 | 20.5 | 0.267 |  |
| Modality assessment | Clinical examination | 10 | 0.88 (0.77-0.99) | 0.040 | 39.9 | 0.092 | 0.004 |
|  | Self-reported | 1 | 0.56 (0.34-0.93) | 0.025 | 0.0 | 1.000 |  |
| *Alzheimer's Disease* | |  |  |  |  |  |  |
| Region | European countries | 4 | 0.92 (0.78-1.10) | 0.362 | 0.0 | 0.412 | 0.062 |
|  | Asian countries | - | - | - | - | - |  |
|  | Western countries | 3 | 0.60 (0.40-0.91) | 0.016 | 17.6 | 0.297 |  |
| Design | Prospective^&^ |  |  |  |  |  |  |
|  | CS/CC^&&^ |  |  |  |  |  |  |
| Year of publication | <2018 | 5 | 0.73 (0.55-0.97) | 0.032 | 45.6 | 0.118 | 0.130 |
|  | ≥2018 | 2 | 1.00 (0.75-1.32) | 0.991 | 0.0 | 0.773 |  |
| Follow-up, years | <10^#^ | 6 | 0.78 (0.61-1.00) | 0.054 | 40.6 | 0.134 | 0.322 |
|  | ≥10^##^ | 1 | 0.97 (0.69-1.37) | 0.862 | 0.0 | 1.000 |  |
| Sample size | <5000 | 4 | 0.71 (0.49-1.03) | 0.074 | 45.2 | 0.140 | 0.305 |
|  | ≥5000 | 3 | 0.90 (0.70-1.15) | 0.398 | 27.7 | 0.251 |  |
| Average age of participants, years | <70 | 2 | 0.98 (0.80-1.21) | 0.869 | 0.0 | 0.926 | 0.074 |
|  | ≥70 | 5 | 0.71 (0.54-0.95) | 0.018 | 26.2 | 0.247 |  |
| Modality assessment | Clinical examination | 7 | 0.83 (0.67-1.01) | 0.065 | 33.8 | 0.170 | 0.074 |
|  | Self-reported | - | - | - | - | - |  |
| *Cognitive impairment/decline* | |  |  |  |  |  |  |
| Region | European countries | 4 | 0.81 (0.70-0.95) | 0.008 | 0.0 | 0.847 | 0.223 |
|  | Asian countries | 5 | 0.92 (0.84-1.01) | 0.080 | 0.0 | 0.713 |  |
|  | Western countries | 4 | 0.78 (0.61-0.98) | 0.033 | 86.6 | 0.000 |  |
| Design | Prospective^&^ |  |  |  |  |  |  |
|  | CS/CC^&&^ |  |  |  |  |  |  |
| Year of publication | <2018 | 6 | 0.88 (0.78-0.99) | 0.032 | 0.0 | 0.450 | 0.644 |
|  | ≥2018 | 7 | 0.84 (0.73-0.96) | 0.010 | 74.7 | 0.001 |  |
| Follow-up, years | <10^#^ | 6 | 0.80 (0.62-1.02) | 0.067 | 75.6 | 0.001 | 0.447 |
|  | ≥10^##^ | 7 | 0.88 (0.82-0.94) | 0.000 | 6.7 | 0.377 |  |
| Sample size | <5000 | 8 | 0.82 (0.67-1.00) | 0.049 | 67.3 | 0.003 | 0.506 |
|  | ≥5000 | 5 | 0.88 (0.82-0.95) | 0.002 | 29.0 | 0.228 |  |
| Average age of participants, years | <70 | 5 | 0.91 (0.83-0.99) | 0.037 | 40.8 | 0.149 | 0.170 |
|  | ≥70 | 8 | 0.79 (0.67-0.94) | 0.007 | 60.1 | 0.014 |  |
| Modality assessment | Clinical examination | 2 | 0.91 (0.72-1.15) | 0.438 | 0.0 | 0.534 | 0.170 |
|  | Self-reported | 11 | 0.84 (0.76-0.94) | 0.002 | 64.1 | 0.002 |  |

Supplementary Table 9. Comparison of the risk of specific cognitive outcomes between individuals reporting the highest versus the lowest fish intake by APOE genotype strata (carrying APOE ε4 allele vs. possessing ε2 or ε3 alleles).

| Outcome | n. studies | RR (95% CI) | *P* | *I^2^* | *P_heter_* |
| --- | --- | --- | --- | --- | --- |
| *APOE ε4 allele* |  |  |  |  |  |
| Dementia | 3 | 1.26 (0.78-2.01) | 0.344 | 3.4 | 0.355 |
| Alzheimer's Disease | 1 | 1.20 (0.43-3.34) | 0.727 | - | - |
| Cognitive impairment/decline | 1 | 0.18 (0.05-0.63) | 0.007 | - | - |
| *APO ε2 or ε3 alleles* |  |  |  |  |  |
| Dementia | 3 | 0.77 (0.58-1.03) | 0.078 | 0.0 | 0.648 |
| Alzheimer's Disease | 1 | 1.07 (0.59-1.94) | 0.823 | - | - |
| Cognitive impairment/decline | 1 | 0.74 (0.42-1.30) | 0.296 | - | - |

Supplementary Figure 1 Flow chart of study selection process.


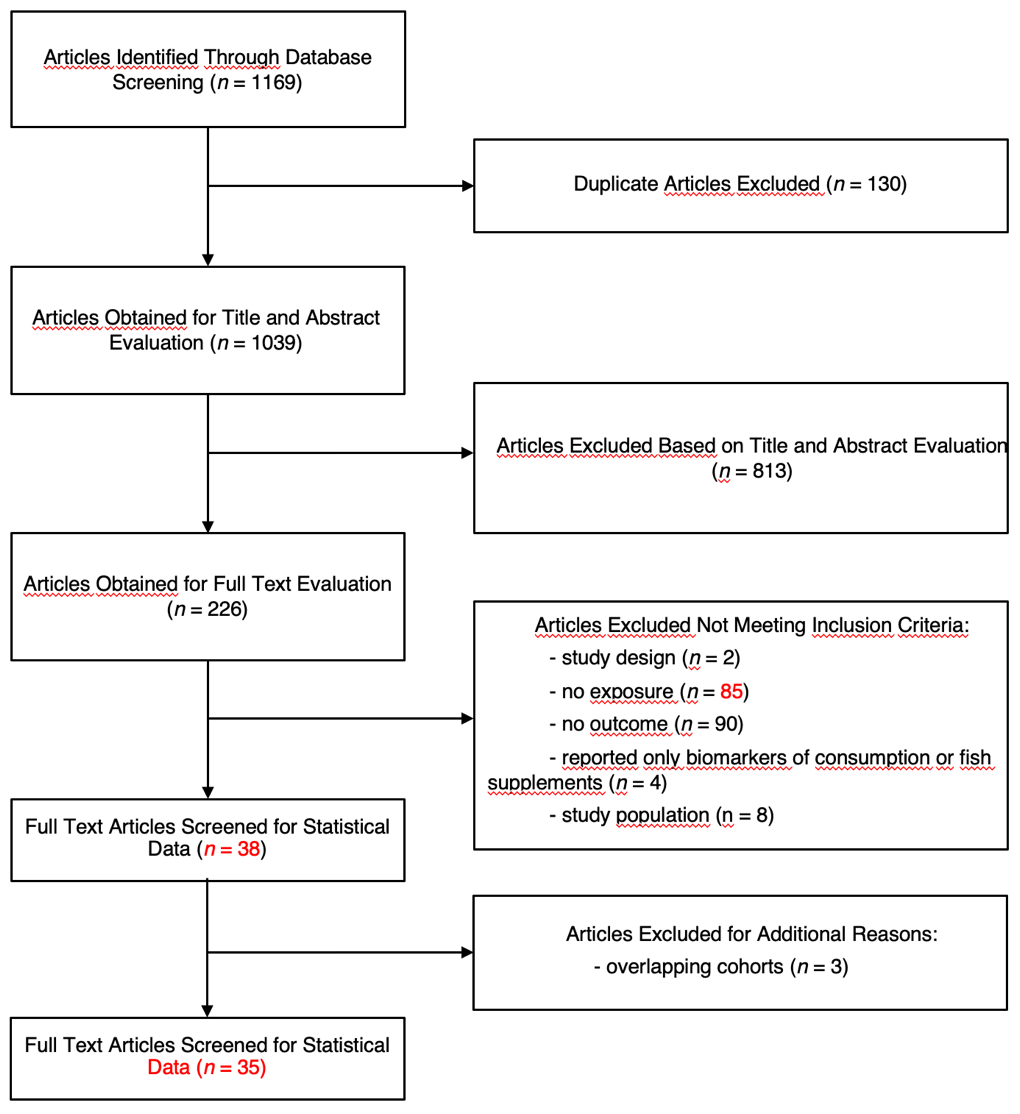


Supplementary Figure 2 Sensitivity analysis for the comparison of the risk of specific cognitive outcomes between individuals reporting the highest versus the lowest fish intake with exclusion of one study at the time.

***Dementia***


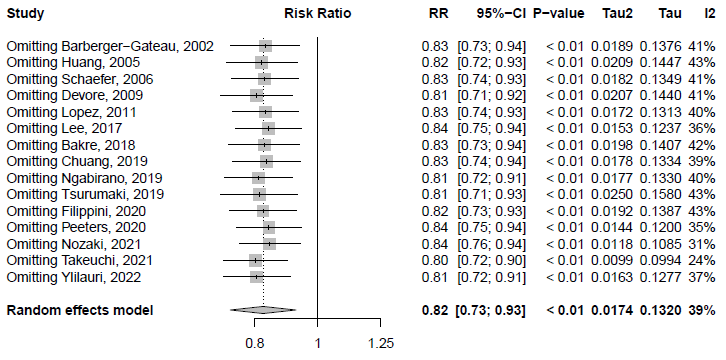


***Alzheimer’s disease***


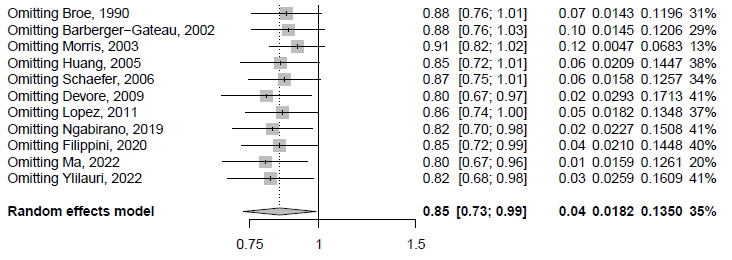


***Cognitive impairment/decline***


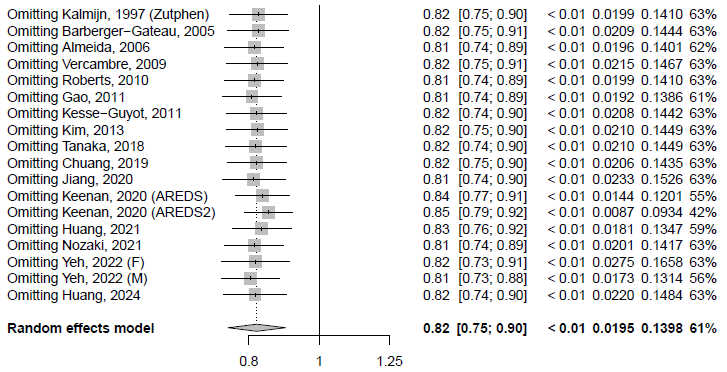


Supplementary Figure 3. Funnel plots of the comparison of the risk of specific cognitive outcomes between individuals reporting the highest *vs*. the lowest fish intake.


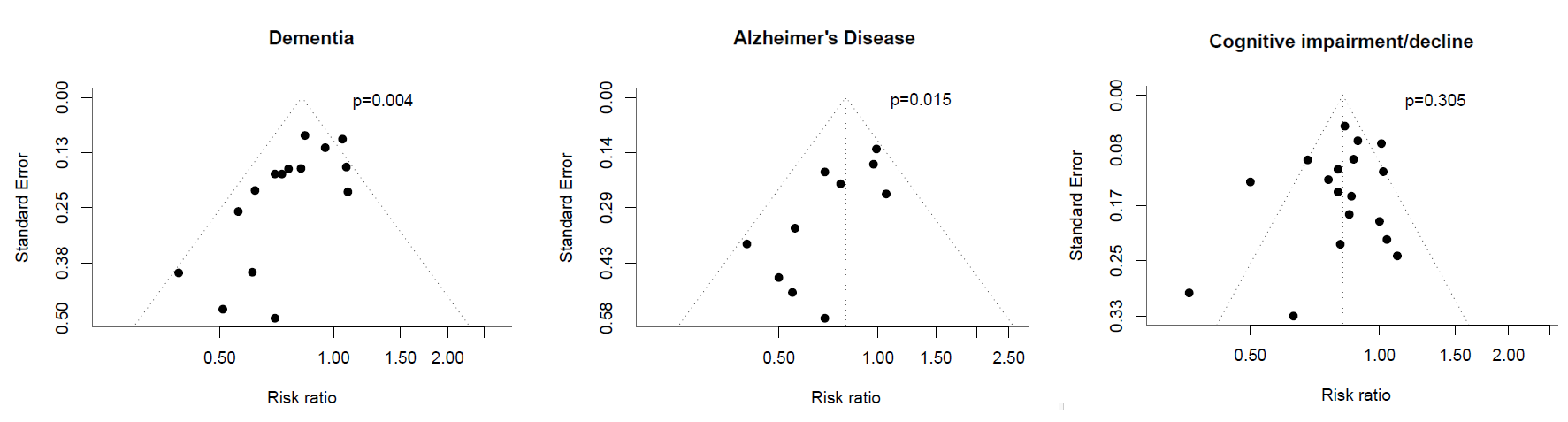


Supplementary Figure 4. Trim and fill analysis of the funnel plots of the comparison of the risk of specific cognitive outcomes between individuals reporting the highest versus the lowest fish intake.


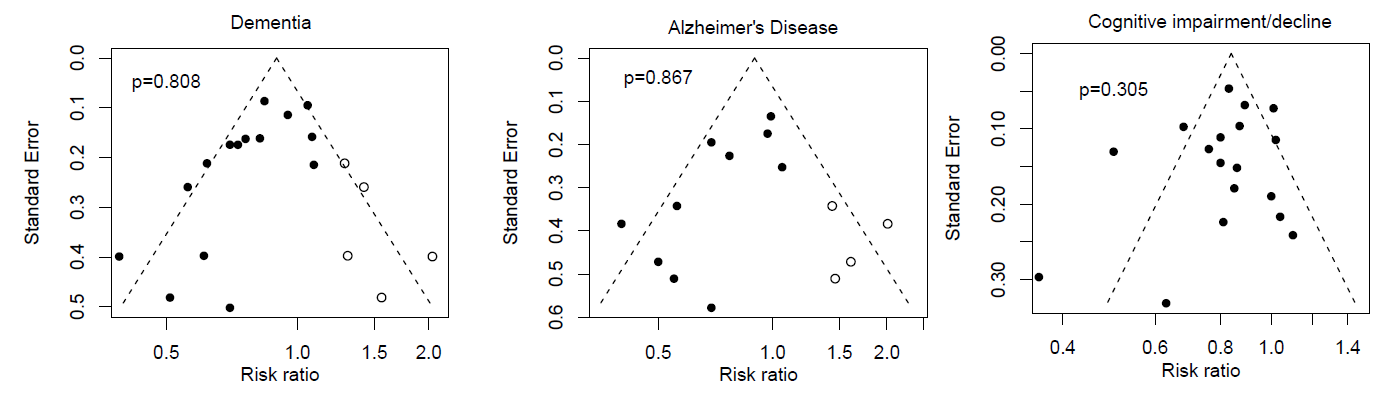


Supplementary Figure 5 Comparison of the risk of specific cognitive outcomes between individuals reporting the highest versus the lowest fish intake by APOE genotype strata (carrying APOE ε4 allele vs. possessing ε2 or ε3 alleles).


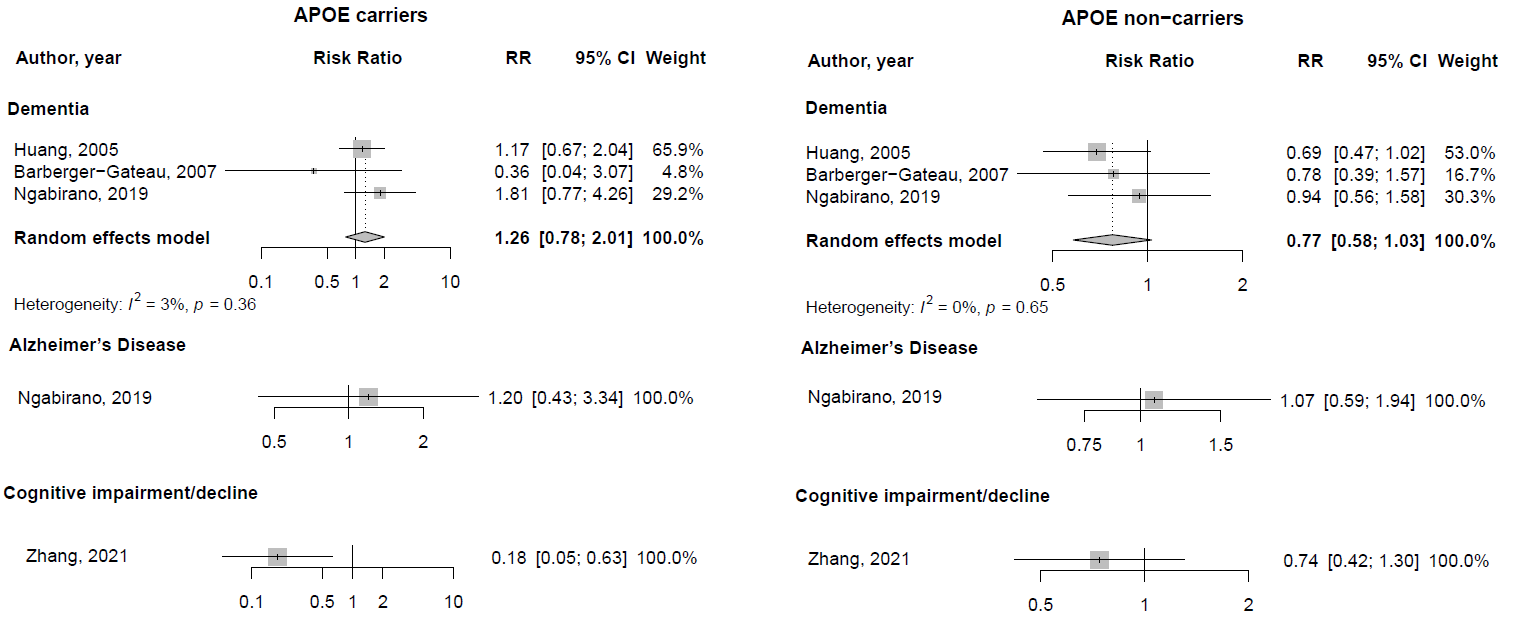

Supplement: Supplementary file 1 — Supplementary Material 1 [file 40520_2024_2823_MOESM1_ESM.docx]
